# Hawthorn Vinegar in Health with a Focus on Immune Responses

**DOI:** 10.3390/nu16121868

**Published:** 2024-06-14

**Authors:** Nilay Seyidoglu, Deniz Karakçı, Buket Bakır, Seydi Yıkmış

**Affiliations:** 1 Department of Physiology, Faculty of Veterinary Medicine, Tekirdag Namik Kemal University, 59030 Tekirdag, Türkiye; 2 Department of Biochemistry, Faculty of Veterinary Medicine, Tekirdag Namik Kemal University, 59030 Tekirdag, Türkiye; dkarakci@nku.edu.tr; 3 Department of Histology and Embryology, Faculty of Veterinary Medicine, Tekirdag Namik Kemal University, 59030 Tekirdag, Türkiye; buketbakir@nku.edu.tr; 4 Department of Food Technology, Tekirdag Namik Kemal University, 59030 Tekirdag, Türkiye

**Keywords:** hawthorn, hawthorn vinegar, rat, ultrasound-treated

## Abstract

Background: The hawthorn fruit is an interesting medicinal plant that has several biological features, especially related to anti-inflammatory, antioxidant and immune-modulating actions, and boosting general health. In this study, we aimed to clarify the immunological effects of hawthorn vinegar on immunity and general health. We also focused on three different production processes to improve the antioxidant activity of hawthorn vinegar (2) Methods: In the study, besides the traditional production of hawthorn vinegar (N), thermal pasteurization (P) and ultrasound (U) techniques were applied to vinegars. A total of 56 female adult Wistar albino rats were randomly allocated into seven groups; Control, N0.5 (regular vinegar; 0.5 mL/kgbw), N1 (regular vinegar; 1 mL/kgbw), P0.5 (pasteurized vinegar; 0.5 mL/kgbw), P1 (pasteurized vinegar; 1 mL/kgbw), U0.5 (ultrasound treated vinegar; 0.5 mL/kgbw), and U1 (ultrasound treated vinegar; 1 mL/kgbw). Vinegars were administered by oral gavage daily. The average weight gains, body mass index, and blood hematological parameters were measured, and the Neutrophil Lymphocyte ratio was calculated. The plasma IL-1β and TNF-α values, and MDA, IL-1β and TNF-α values of intestinal tissue, were determined. Also, the streptavidin–biotin–peroxidase complex method was applied to determine the expressions of TNF-α and IL-1β in duodenum. (3) Results: There was a decreasing tendency in the average weight gains in all vinegar groups compared to the control group. In addition, there was an increase in NL ratio in all vinegar groups, although not significant. There were no statistical differences among all vinegar groups, although decreases were observed in plasma IL-1β. Also, the plasma TNF-α values showed slight increases in high-dose-of-vinegar groups (N1, P1 and U1), although not significant. In addition, the intestinal tissue IL-1β value tended to increase in groups N0.5, N1 and P0.5, while it tended to decrease in P1, U0.5 and U1. On the other hand, there were slight increases in the TNF-α values of intestinal tissue in all groups compared to control, although these were not significant. Furthermore, the intensive expressions of TNF-α and IL-1β were determined in groups U0.5 and U1. (4) Conclusions: The results suggest that either high doses or ultrasound applications of hawthorn vinegar have positive effects on intestinal health, boosting immunity and general health.

## 1. Introduction

There is a complex interaction that takes place between nutrition and immunity. Nutrient sources such as protein, vitamins or minerals in either large or small amounts may cause metabolic abnormalities. These disturbances can affect and modify the immunity and health. Researchers have addressed the interaction among nutrient sources, immune responses and health over the years. However, the mechanisms underlying this association remain poorly understood [1,2,3,4,5]. It has been found reviewed that cytokines act as the hormones of the immune system through metabolic responses. In addition, metabolic and catabolic mechanisms of cytokines can change the nutrient status in blood and the intestinal system. On the other hand, another mechanism is the nutrient-induced changes of cytokines under normal and pathological conditions. This mechanism can help to design dietary modifications against diseases, and to protect general health. Therefore, it was suggested that diets including adequate nutrients can maintain the health status of an organism [6].

The Food and Drug Administration (FDA) announced the definition of several vinegars [7]. Vinegar is not only a natural drink, but also a functional health food. Researchers reported that vinegar can prevent several diseases, especially obesity, cancer and metabolic diseases [8,9]. In addition, it was determined that vinegar can play an important role in digestion, improving the gut microbiome as a result of the bioactive compounds, including amino acids, proteins, carbohydrates, polyphenols and other antioxidants [8,9,10]. As a result of these contents, vinegar has several important effects, such as immune-enhancing, anti-oxidative and antidiabetic [11]. It was reported that these compounds, especially polyphenols, in vinegar can modulate the gut composition, and boost the intestinal health [12,13]. Nevertheless, the methods used for vinegar production are also important as they enhance the bioactive components of vinegars [14,15]. It was determined that the functional properties of vinegar produced by conventional methods may be higher than those of industrial vinegars [14,15]. It also provides the possibility to get better-tasting vinegar. In this context, ultrasound pasteurization, a new non-thermal technology, is currently becoming more popular. In addition, significant microbial inactivations, minimal impacts on the deterioration of quality parameters, and improvements in functionalities with ultrasound treatment applied to liquid foods have been reported [16,17,18].

There are several kinds of vinegar that have been produced from several fruits and plants. The one attracting the most interest in recent years is hawthorn vinegar [8,9,18,19]. Hawthorn fruit has been known of since ancient times in a large area of the world [20,21]. The fruit belongs to the rose family, which has 280 varieties in its genus. In addition, the leaves and flowers of this fruit contain rich, biologically active compounds, such as phenolic, flavonoids, terpenoids, etc. [22]. Hawthorn fruit plays important roles in several metabolisms, especially appetite, blood glucose, lipid metabolism, and immunity, due to its antioxidant, anti-inflammatory and immune efficiencies [22]. Liu et al. [23] reported that the polyphenol and flavonoid contents of hawthorn fruit can prevent and treat inflammation. They observed that flavonoids have anti-inflammatory effects that limit TNF-α in the intestinal barrier. Also, Elango and Devaraj [24] found that the proinflammatory cytokines, especially IL-1β, IL-6, and TNF-α, can alleviate pro-inflammatory immune responses in rats. It has also been suggested that a possible mechanism of its action could be related to hawthorn polyphenol extracts. In addition, according to the literature and reviews, hawthorn vinegar has received increasing attention due to its positive effects on hyperglycemia, dyslipidemia, and lowering blood pressure [22]. However, to our knowledge, there is limited information available regarding the effects of hawthorn vinegar on immunity and health.

Prophylactic approaches have been sought to protect human health against stress, as well as disease outbreaks. In particular, nowadays, people tend to search for nutrient sources to protect and support their health against diseases, especially pandemic diseases, influenza and other metabolic diseases. Also, researchers have studied medicinal herbs, vinegars, and plant extracts for this purpose. In this sense, in this study, we aimed to evaluate the effects of hawthorn vinegar produced via three different methods on plasma and intestinal tissue immune parameters, as well as immunohistochemical changes in the intestinal tissue of adult rats. Also, we sought to determine the efficiency of the method of producing hawthorn vinegar via ultrasound in improving the effects of bioactive components on immunity and health.

## 2. Materials and Methods

### 2.1. Animals and Feeding

The experiments were performed in accordance with the guidelines provided by the National Institute of Health for Animal Research and approved with ethical permits from the University Animal Experiments Local Ethics Committee (Ethical Approval No. 2021-609, approved on 6 April 2021). In the study, 56 female Wistar albino rats, weighing 230–270 g and aged 6–7 months, were allocated to 7 experimental groups. The rats were given ad libitum access to commercial pelleted rodent diet and tap water. Hawthorn vinegar was given to the rats by oral gavage daily throughout the 45 trial days. The groups were set out as below:

1. Group—Control (basal diet);
2. Group—N0.5 (0.5 mL/kg untreated hawthorn vinegar daily);
3. Group—N1 (1 mL/kg untreated hawthorn vinegar daily);
4. Group—P0.5 (0.5 mL/kg thermal pasteurized hawthorn vinegar daily);
5. Group—P1 (1 mL/kg thermal pasteurized hawthorn vinegar daily);
6. Group—U0.5 (0.5 mL/kg hawthorn vinegar ultrasound treated daily);
7. Group—U1 (1 mL/kg hawthorn vinegar ultrasound treated daily).

### 2.2. Hawthorn Vinegar

Hawthorn fruit (*Crataegus tanacetifolia*) samples were sourced from Bursa/Turkey, and the untreated hawthorn vinegar was produced using the method of conventional vinegar manufacturing. After vinegar production, the vinegars were processed by 3 methods of modification [14]. The first product was the vinegar without any treatment (Group N0.5 and N1). The second group’s method was thermal pasteurization (Groups P0.5 and P1). The third group’s methods were the response surface method and ultrasound treatment (Groups U0.5 and U1).

### 2.3. Determination Bioactive Compounds of Vinegar

The total phenolic content of samples was analyzed by Folin–Ciocalteu method [25]. All analyses were performed in triplicate. The absorbance was measured on a UV–Vis spectrophotometer (SP-UV/VIS-300SRB, Spectrum Instruments, Melbourne, Australia).

The total flavonoid contents of samples were determined by the colorimetric technique [26], and the concentrations were calculated colorimetrically using a UV spectrophotometer (Spectrum Instrument, SP-UV/VIS-300SRB, Australia).

The ascorbic acid concentration of the vinegars was calculated by the AOAC 961.27 vitamin preparation and ascorbic acid 2.6 dichlorophenol indophenol-titrimetric methods [27].

The antioxidant capacities of vinegars were evaluated using the CUPRAC and DPPH methods previously described by [28,29,30].

### 2.4. Measurements

The weights of rats were measured at the beginning and the end of the trial, and the average weight gains were determined. The average weight gains, body mass indexes (BMIs) and feed conversation ratios (FCRs) were calculated. Blood samples were obtained by puncturing the heart under isoflurane anesthesia at the end of the study. The blood hematocrit, hemoglobin, counts of erythrocyte and leukocyte, and types of leukocyte (neutrophil, lymphocyte and monocyte) were determined using an automatic blood counter device (Exigo, Istanbul, Türkiye) in the Laboratory of the Experimental Animal Institute at University. Also, the neutrophil lymphocyte ratio (NL ratio) was recorded to evaluate whether the rats were stressed or not. According to the literature, NL ratio may serve as an early warning of the pathological state or processes such as stress, inflammation and psychiatric disorders. The NL ratio was calculated from complete white blood cell counts by dividing the absolute count of neutrophils by that of lymphocytes [31].

The blood samples were centrifuged on the same day at 3000 rpm for 10 min at 4 °C to separate the plasma. The intestinal tissue samples were separated into pieces of appropriate sizes (1 g tissue, 9 mL PBS). Then, samples were placed in PBS (pH 7.4) and adjusted to a ratio of tissue weight (g):PBS (mL) = 1:9 volumes. Then, homogenates were prepared using a homogenizer (Interlab, Istanbul, Türkiye). The tissue homogenates were centrifuged at 5000× *g* for 5 min at 4 °C, and then supernatants were added into microtubes. All blood and tissue samples were stored at −80 °C until the analysis. The Interleukin-1β (IL-1β) and Tumor Necrosis Factor-α (TNF-α) concentrations of plasma and intestinal tissue supernatants were measured using the commercially available rat ELISA kits Rat TNF-α ELISA Kit (Cat. No. 201-11-0765) and Rat IL-1β ELISA Kit (Cat. No. 201-11-0120, (Shanghai Sunred Biotechnology, Shanghai, China), and the assays were performed using a microplate reader (Biotek, Epoch, Winooski, VT, USA).

The duodenum samples were fixed for 24 h in a 10% formalin solution. After that, the tissue samples were dehydrated (ethanol), cleared (xylene), and embedded in paraffin. The paraffin blocks were cut into 5 μm-thick sections on a rotary microtome (LIECA) and stained with Mallory’s modified triple-staining approach to examine the general structure of the duodenum [32]. The streptavidin biotin peroxidase complex (strepABC) method was used to determine the localization of TNF-α and IL-1β immunohistochemically in the duodenum tissues of all groups. Here, 5 μm cross-sections were fixed on the adhesive slides and were subjected to deparaffinization and dehydration. The sections were processed in citrate buffer solution (pH 6.0) for 10 min in a microwave oven at 800 watts. Then, tissues were held in 3% hydrogen peroxide (H_2_O_2_) for 15 min. Blocking solution A was dripped to prevent nonspecific binding by the IHC Kit (Invitrogen-Histostatin Plus Bulk Kit, Waltham, MA, USA). TNF-α primary antibody (ab220210, TNF-α/11721, 1/100 dilution) and IL-1β primary antibody (ab205924, 1/300 dilution) were applied on the sections in a humid environment at ambient temperature for 1 h. Then, secondary antibodies and streptavidin (Thermo Scientific, Ultravision Large Volume Detection System Anti-Polyvalent, HRP, TP-125-BN, and TS-125-HR, Deutsch, Germany) were dripped onto the sections for 30 min. To demonstrate the antibody reaction, the DAB (3.3′-Diaminobenzidine) chromogen solution was added to the cross-sections for 10 min. Then, Gill III hematoxylin was used for the counterstaining and covered with entellan. Negative controls were set up without the primary antibody. The sections were evaluated by two independent observers using the semi quantitative method by taking the degree of staining in the cross-sections as a criterion. Immunoreactive cells were categorized as negative, mild, moderate, or intensive [33,34]. The immunohistochemically prepared samples were photographed and assessed under a light microscope (Olympus BX51, Tokyo, Japan).

### 2.5. Statistical Analyses

Data were expressed as mean ± SEM using Graph Pad Prism Graphical, Statistical package version 5 (30 days demo version). Statistical analysis was performed using ANOVA, followed by significant difference tests to derive comparisons between individual groups. The non-parametric Dunnett Comparison Test was applied to determine differences in variables with 5% levels of significance (*p* < 0.05).

## 3. Results

Hawthorn vinegar is a rich source of flavonoid antioxidants. In the present study, the hawthorn vinegar derived via the ultrasound method had the highest values of total phenolic (116.99 mgGAE/100 mL) and flavonoid (15.89 mgCE/100 mL) compounds, as well as DPPH (62.35%) and CUPRAC (67.39%) levels. However, the antioxidant contents of products derived via the thermal pasteurization processing method (DPHH—54.86%; CUPRAC—60.22%; total flavonoid—13.18 mg CE/100 mL; total phenolic—104.22 mgGAE/100 mL) are lower than those of vinegar derived via normal processing (DPHH—57.39%; CUPRAC—63.55%; flavonoid—14.22 mg CE/100 mL; total phenolic—110.58 mgGAE/100 mL).

**Figure 1:**
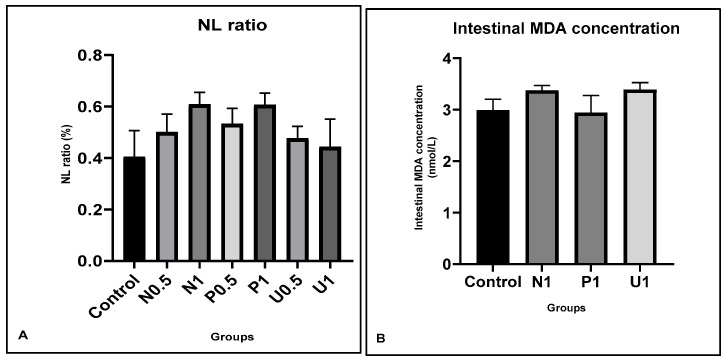
Effects of hawthorn vinegar over 45 days on blood neutrophil/lymphocyte ratio (NL) and intestinal malondialdehyde (MDA) parameters in all groups, to determine if nutritional stress occurred. (**A**) NL ratio in all groups; (**B**) intestinal MDA. All data are presented as the mean ± SE (n = 8).

The average body weight gains, body mass indexes (BMI, kg/m^2^) and feed conversion ratio (FCR, %) are shown in Table 1. Although there were no statistical differences, the average of body weight gains (g) decreased in groups N0.5, N1, P1 and U1, while they increased in groups P0.5 and U0.5 compared to control group. Also, the FCR decreased in groups N0.5 and U1, while it increased in groups N1, P0.5, P1 and U0.5 compared to control, although not significantly. However, there were no differences in body mass indexes among all groups. In addition, the hematological parameters are shown in Table 2. The hemogram parameters were found at their reference values among all vinegar groups compared to the control. Also, for clarity, the nutritional stress, blood NL ratio and tissue malondialdehyde (MDA) parameters are shown in Figure 1A,B. There was a tendency of increase in the NL ratio and tissue MDA values in the high-vinegar groups (N1, P1 and U1) compared to the control group, although this was not significant.

**Figure 2:**
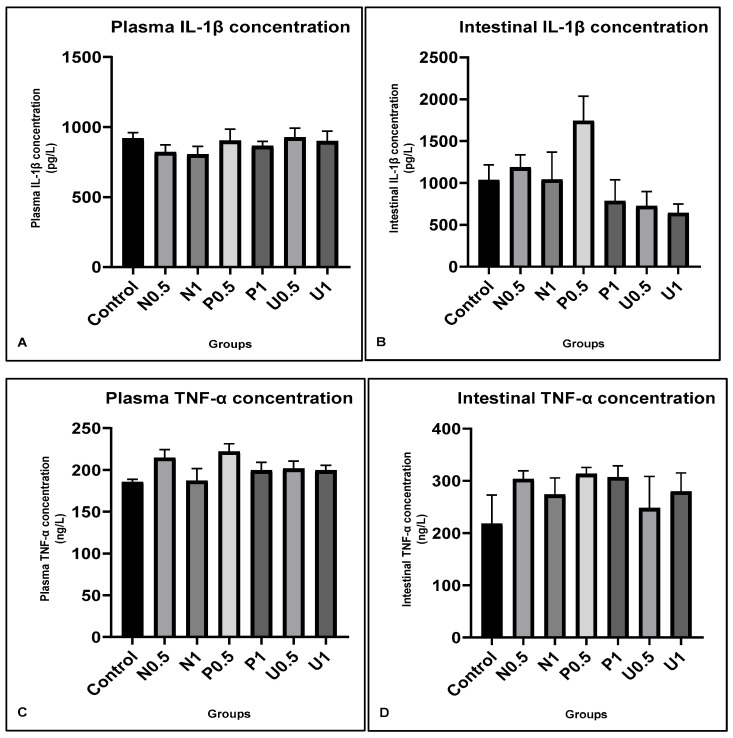
Effects of hawthorn vinegar over 45 days on plasma and intestinal tissues’ IL1 and TNF alfa concentrations in all groups. (**A**) Plasma IL-1β con. (**B**) Intestinal tissue IL-1β con. (**C**) Plasma TNF-α con. (**D**) Intestinal tissue TNF-α con. All data are presented as the mean ± SE (n = 8).

The plasma and intestinal tissues’ IL-1β and TNF-α parameters in experimental animals are shown in Figure 2. There were no statistical changes, although decreases were observed in plasma IL-1β in all vinegar groups (Figure 2A). However, although non-significant, the plasma TNF-α values showed slight increases in the high-dose vinegar groups (N1, P1 and U1), shown in Figure 2C. In addition, the intestinal tissue’s IL-1β showed a tendency to increase in groups N0.5, N1 and P0.5, while it decreased in P1, U0.5 and U1 (Figure 2B). On the other hand, there were slight increases in the intestinal tissues’ TNF-α in all groups compared to the control, although these were not significant (Figure 2D).

**Figure 3:**
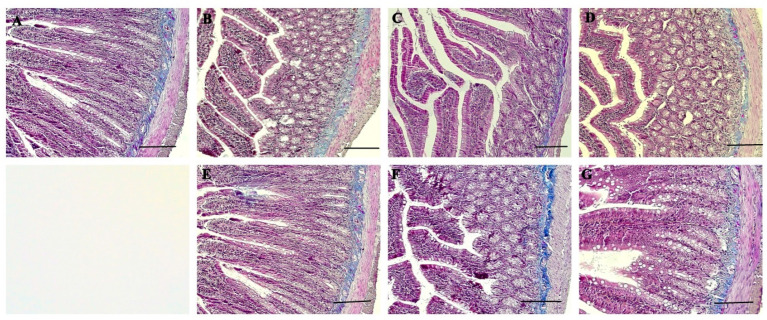
Microphotography of rat duodenum. Control group (**A**), N0.5 group (**B**), P0.5 group (**C**), U0.5 group (**D**), N1 group (**E**), P1 group (**F**), U1 group (**G**). Triple staining, bars = 200 μm.

**Figure 4:**
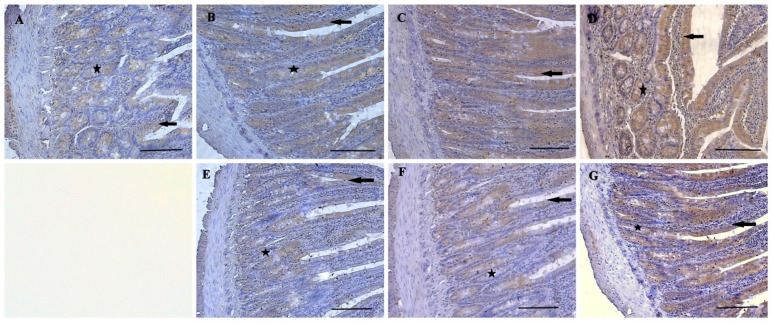
TNF-α expression in the rat duodenum. Control group (**A**), N0.5 group (**B**), P0.5 group (**C**), U0.5 group (**D**), N1 group (**E**), P1 group (**F**), U1 group (**G**). Villus epithelial cell (arrow), crypt epithelial cell (star), immunohistochemical staining, bars = 200 μm.

**Figure 5:**
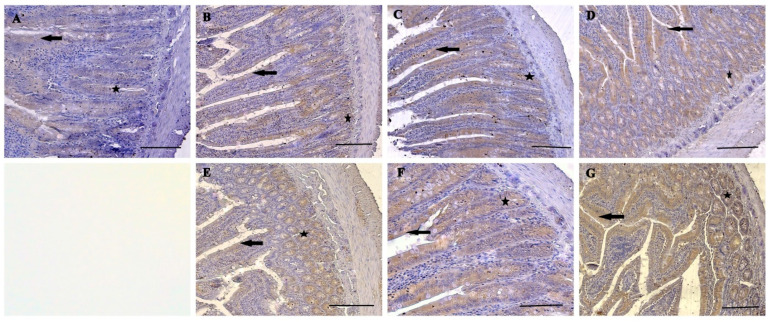
IL-1β expression in the rat duodenum. Control group (**A**), N0.5 group (**B**), P0.5 group (**C**), U0.5 group (**D**), N1 group (**E**), P1 group (**F**), U1 group (**G**). Villus epithelial cell (arrow), crypt epithelial cell (star), immunohistochemical staining, bars = 200 μm.

The normal histological structure of duodenum is shown in Figure 3. TNF-α and IL-1β immunoreactivities were observed in the duodenum of all groups. However, while moderate TNF-α expression was observed in villus epithelial cells, and crypt epithelial cells were observed in the N0.5, P0.5, N1, and P1 groups (Figure 4B,C,E,F), intensive expressions were detected in the U0.5 group and the U1 group (Figure 4D,G). In the control group, mild-intensity TNF-α expression was detected in villus and crypt epithelial cells (Figure 4A). Nevertheless, the villus epithelial cells and crypt epithelial cells in the N0.5, P0.5, U0.5, N1, and P1 groups showed moderate IL-1β expression (Figure 5B–F), and intensive IL-1β expression was seen in the U1 group (Figure 5G). Also, mild IL-1β expression was observed in the duodenum of the control group (Figure 5A).

## 4. Discussion

Scientific studies have indicated that dietary nutrient intake is important to strengthening the immune system and immune response. In the last few years, several papers have addressed a huge number of aspects of nutrition in relation to pandemic and metabolic diseases. Also, due to climate changes and food crises, scientists have been working to intensify the efficiencies of nutrient sources. Related to this, people have also tended to consume natural products, which have positive effects on the metabolism and immune system. Among these nutrient sources, hawthorn fruit, its extracts and its vinegars have more beneficial antioxidant properties. Hawthorn is a traditional fruit, especially prevalent in Europe, Asia and China. There are several industrial products derived from this fruit in use by people, such as juice and vinegar. However, there have been limited studies into hawthorn vinegar and its benefits. The current study outlines the protective effects on growth and immunity of hawthorn vinegar produced via ultrasound processing and thermal pasteurization methods. In addition, the results in this study suggest that the highest immune activities were found in vinegar produced by the ultrasound method.

Nutritional and pharmacological studies have shown that hawthorn fruit improves intestinal flora and gastrointestinal function [22]. It has been noted that, due to its richness of bioactive components, it acts as a prebiotic that mediates the gut microbiota [22]. In particular, polysaccharides in hawthorn fruit and extracts play essential roles in the gastrointestinal tract, blood and metabolism [12,35,36]. Several mechanisms have been reported explaining the correlation between vinegar consumption and body weight. The most widely known effects are hunger reduction and decreased effects of food intake [37]. Similarly, in our study, the average weight gains show a decreasing tendency in groups with high doses of hawthorn vinegar (N1—0.50 ± 2.59, P1—0.88 ± 1.75, and U1—1.38 ± 1.69) compared to the control group (2.75 ± 2.97). In addition, the body mass index values (BMI) of all groups were found to be similar among all groups. Also, the feed conservation ratio (FCR) tended to increase in groups with high doses of vinegar (N1—0.56 ± 0.17, P1—0.40 ± 0.14 and U1—0.40 ± 0.08) compared to the control group (0.36 ± 0.11). The changes in average weight gains, FCR and BMI may be due to the daily consumption of vinegar and its dose-dependency. These parameters are known to vary, depending on diet and nutrient sources. However, it can be said that daily exposure to hawthorn vinegar can lead to balanced weight gain. Besides this, the hematological parameters can be useful to general health assessments. Rababa’h et al. [38] found that there were no differences in the hematological parameters of groups fed the hawthorn fruit. Also, Lis et al. [36] determined that hawthorn fruit promotes the proliferation of splenocytes, and increases the numbers of hematological cells. These findings are in line with our results. In our study, there were no differences in any of the hematological parameters in any vinegar groups compared to the control group. This means that both low and high doses of hawthorn vinegar can be considered as nutrient sources benefitting general health.

Optimal nutrition is essential to improving the immune system, general health, and quality of life. The immune system can be evaluated by the antioxidant and immune responses of the plasma and intestinal tissue. Although various dietary and bacterial stimuli affect the intestines every day, it is not always possible for immune responses to develop. Therefore, optimal nutrition is necessary to the formation and balancing of the immune responses. The blood’s cells and proteins, as well as cytokines, are the most important components of the immune system, as is well known. As for the immune responses, IL-1β and TNF-α should be assessed to ensure homeostasis [39]. In addition, the cytokines IL-1β and TNF-α are known as proinflammatory proteins that arise in stressful conditions [40,41]. In our study, slight decreases were seen in plasma IL-1β with high doses of hawthorn vinegar (N1—804.13 ± 57.20; P1—865.28 ± 32.49 and U1—898.19 ± 72.79) compared to the control (917.44 ± 42.49). In addition, plasma TNF-α values showed slight increases in groups N1 (186.85 ± 17.72), P1 (199.31 ± 9.71) and U1 (199.32 ± 6.04) compared to the group control (185.29 ± 3.54), although these were not significant. Similar to our results, Xia et al. [42] reported that polyphenols in vinegars could reduce the plasma and tissue IL-1β and TNF-α values in diabetic rats. Also, Hu et al. [43] determined that hawthorn fruit can reduce the plasma and tissue’s IL-1β and TNF-α values. They suggested that the hawthorn fruit may effectively relieve inflammatory responses.

The regulation of intestinal microbiota has been associated with epithelial barrier functions and intestinal immune cell activation. The inflammatory process taking place in all layers of the gut wall is regulated by neuro–immune cooperation [44]. It is accepted that TNF-α and IL-1β play roles in a spectrum of physiological processes related to inflammation and homeostasis in intestinal tissues. The TNF-α level in the intestinal layer is dependent on physiological conditions. Nevertheless, IL-1β was observed to be one of the more important cytokines playing a role in the cytokine network, and thereby controlling the most important functions of the immune system [45]. In our study, we saw an intensive expression of TNF-α in duodenal cells in groups U0.5 and U1. The intensive IL-1β expression in group U1 could be related to the activation of cellular immunity. According to this, increases in TNF-α can be confirmed to occur under nutritional stress, mainly resulting from greater duodenal inflammation. On the other hand, the higher IL-1β expressions in the U1 group could be explained by the protective effects of vinegar’s polyphenols on oxidative damage. These findings also agree well with the results for blood and tissue concentrations in the study. However, the signaling pathways, specific mechanisms and functions of TNF-α and IL-1β must be investigated in order to guide medical actions.

Moreover, in our study, the MDA values in the intestinal tissue were slightly increased in high-dose hawthorn vinegar groups (N1—3.39 ± 0.14; P1—3.38 ± 0.10 and U1—3.18 ± 0.11) compared to the control (2.99 ± 0.21). In addition, the plasma neutrophil/lymphocyte (NL) ratio tended to increase in all high-vinegar groups compared to the control group (0.40 ± 0.10, 0.61 ± 0.05, 0.61 ± 0.05 and 0.44 ± 0.11; control, N1, P1 and U1, respectively). The increases in NL ratio, MDA and TNF-α may be due to the stressful situation developing in organisms that is caused by nutrition, especially in the high-dose vinegar groups P1 and U1. Also, the slight reductions in IL-1β values coincided with the activation of immunity in both intestinal tissue and blood in groups P1 and U1. These results show that, whether one uses the high-dose or ultrasound method, hawthorn vinegar application could improve the intestinal tissue’s defense against nutritional stress, as well as general health. Also, it could be suggested that by increasing the effectiveness of antioxidant components in hawthorn vinegar via the ultrasound method, the antioxidant functions of vinegar can be made more effective.

Ultrasound technology is an important innovation in relation to food preservation. It is a process that minimizes the losses of bioactive contents [46]. In particular, phenolic and flavonoid compounds, and antioxidant DPHH and CUPRAC ratios can be increased by this method. Demirok et al. [47] studied tangerine juice subjected to ultrasound technology, and they derived greater total antioxidant and food safety levels. In our study, the DPHH and CUPRAC ratios, and antioxidant compounds such as phenolics, were found to be higher when subjected to ultrasound technology compared to other methods. Furthermore, the results were stronger in the high dose of ultrasound vinegar group (U1) compared with other groups. The findings of the ultrasound method denote acceptable effects on immunity and general health. Fruit vinegars have a long shelf life and abundant antioxidant compounds, which is why they are preferred by people.

## 5. Conclusions

Nutrient sources, as pointed out by scientists, have key effects on the intestinal system, immunity and the metabolism. The well-recognized impact of nutrition on intestinal flora elucidates the role of nutrition in improving health. People have been attracted to nutrient sources because of the continuing rise in the global population, which is threatening biodiversity, causing water pollution and leading to food crises. Hawthorn fruit is an economically crucial fruit that is produced in a wide area of the world, and it also contains important bioactive compounds such as phenolics, organic acids, vitamins and minerals. Hawthorn vinegar, as well as the fruit, has several important effects on general health, especially acting as an antioxidant and immune modulator. However, it is also known that, when ultrasound technology is applied, vinegar production has positive effects on antioxidant efficiency, with impacts on health.

The findings of the study indicate that parallel analyses of blood and intestinal tissue yielded the sought information. Slight decreases in IL-1β and increases in TNF-α in both plasma and intestinal tissue were determined in high-dose hawthorn vinegar groups. Most notably, it was found that a 1 mL/kg dose of hawthorn vinegar, along with the utilization of the ultrasound method, offers more beneficial outcomes than other vinegars. As a result, we suggest that hawthorn vinegar has important remedial effects that may boost immune status and health.

## Figures and Tables

**Table 1:** Effects of all groups of hawthorn vinegar after 45 days on some growth parameters.

Parameters	Control	N0.5	N1	P0.5	P1	U0.5	U1
Average weight gain (g)	2.75 ± 2.97	1.25 ± 1.24	0.50 ± 2.59	3.75 ± 1.71	0.88 ± 1.75	4.38 ± 1.58	1.38 ± 1.69
Body mass index (BMI, kg/m^2^)	0.15 ± 0.005	0.15 ± 0.003	0.15 ± 0.003	0.15 ± 0.004	0.15 ± 0.004	0.15 ± 0.003	0.15 ± 0.003
Feed conversion ratio (FCR, %)	0.36 ± 0.11	0.25 ± 0.09	0.56 ± 0.17	0.51. ± 0.38	0.40 ± 0.14	0.48 ± 0.13	0.40 ± 0.08

All data are presented as the mean ± SE (n = 8).

**Table 2:** Effects of hawthorn vinegar over 45 days on some important hemogram parameters for all groups.

Parameters	Control	N0.5	N1	P0.5	P1	U0.5	U1
Hematocrit (%)	29.74 ± 1.64	32.76 ± 3.23	26.75 ± 1.52	28.46 ± 0.98	29.24 ± 2.06	26.81 ± 1.66	28.39 ± 1.59
Hemoglobin (g/dL)	12.96 ± 0.66	14.26 ± 1.35	11.59 ± 0.64	12.33 ± 0.40	12.58 ± 0.88	11.75 ± 0.68	12.36 ± 0.64
Erythrocytes (10^6^/mL)	6.10 ± 0.34	6.84 ± 0.65	5.59 ± 0.31	5.91 ± 0.18	6.06 ± 0.38	5.57 ± 0.32	5.96 ± 0.31
Leucocytes (10^3^/mL)	2.64 ± 0.52	3.56 ± 0.77	2.73 ± 0.56	2.30 ± 0.35	2.99 ± 0.51	1.69 ± 0.22	1.69 ± 0.24
Neutrophils (%)	26.40 ± 5.45	32.40 ± 3.20	37.20 ± 1.83	34.00 ± 2.81	37.00 ± 1.84	31.20 ± 2.17	25.80 ± 4.69
Lymphocytes (%)	71.00 ± 5.16	66.60 ± 2.71	61.80 ± 1.77	64.80 ± 2.20	61.60 ± 1.69	66.20 ± 2.08	71.80 ± 4.93
Eosinophils (%)	2.25 ± 0.48	2.00 ± 1.00	1.25 ± 0.25	1.33 ± 0.33	2.00 ± 1.00	2.00 ± 0.32	2.00 ± 0.71
Monocytes (%)	1.00 ± 0.0001	1.00 ± 0.00001	-	2.00	1.00 ± 0.00001	1.00	1.00 ± 0.00001
Basophils (%)	1.00	-	-	-	1.00	1.00 ± 0.00001	-
Neutrophil/lymphocyte ratio (NL ratio, %)	0.40 ± 0.10	0.50 ± 0.07	0.61 ± 0.05	0.53 ± 0.06	0.61 ± 0.05	0.48 ± 0.05	0.44 ± 0.11

All data are presented as the mean ± SE (n = 8).

## Data Availability

The original contributions presented in the study are included in the article; further inquiries can be directed to the corresponding author.
